# Associations between serum albumin level trajectories and clinical outcomes in sepsis patients in ICU: insights from longitudinal group trajectory modeling

**DOI:** 10.3389/fnut.2024.1433544

**Published:** 2024-07-19

**Authors:** Xin Tie, Yanjie Zhao, Ting Sun, Ran Zhou, Jianbo Li, Jing Su, Wanhong Yin

**Affiliations:** ^1^Department of Critical Care Medicine, West China Hospital, Sichuan University, Chengdu, China; ^2^Department of Neurosurgery, West China Hospital, Sichuan University, Chengdu, China

**Keywords:** sepsis, albumin (ALB), group-based trajectory modeling, clinical outcomes, critical care

## Abstract

**Background:**

Sepsis triggers a strong inflammatory response, often leading to organ failure and high mortality. The role of serum albumin levels in sepsis is critical but not fully understood, particularly regarding the significance of albumin level changes over time. This study utilized Group-based Trajectory Modeling (GBTM) to investigate the patterns of serum albumin changes and their impact on sepsis outcomes.

**Methods:**

We conducted a retrospective analysis on ICU patients from West China Hospital (2015–2022), employing GBTM to study serum albumin fluctuations within the first week of ICU admission. The study factored in demographics, clinical parameters, and comorbidities, handling missing data through multiple imputation. Outcomes assessed included 28-day mortality, overall hospital mortality, and secondary complications such as AKI and the need for mechanical ventilation.

**Results:**

Data from 1,950 patients revealed four serum albumin trajectories, showing distinct patterns of consistently low, increasing, moderate, and consistently high levels. These groups differed significantly in mortality, with the consistently low level group experiencing the highest mortality. No significant difference in 28-day mortality was observed among the other groups. Subgroup analysis did not alter these findings.

**Conclusion:**

The study identified four albumin trajectory groups in sepsis patients, highlighting that those with persistently low levels had the worst outcomes, while those with increasing levels had the best. Stable high levels above 30 g/L did not change outcomes significantly. These findings can inform clinical decisions, helping to identify high-risk patients early and tailor treatment approaches.

## Introduction

Sepsis is characterized as a systemic inflammatory response syndrome initiated by infection, which predominantly presents as multisystem organ dysfunction. The respiratory, cardiovascular, and renal systems are the most commonly affected (1–3). The high mortality rate associated with sepsis poses a significant challenge to critical care medicine. Research indicates that endothelial damage, instigated by the inflammatory response to sepsis, plays a crucial role in the progression of the condition. This damage is a central pathway contributing to diminished vascular reactivity and heightened capillary permeability, leading to disruptions in oxygen transport and hemodynamics that ultimately may result in organ failure (4–6). The endothelial glycocalyx is a layer composed of substances including albumin and proteoglycans that overlays the endothelial cell surface, is pivotal in maintaining vascular integrity. It is fundamental to the preservation of endothelial function (7).

Albumin plays a crucial role in various physiological processes. These include maintaining plasma colloid osmotic pressure, exhibiting antioxidative and anti-inflammatory activities, regulating acid–base balance, and facilitating the transport, distribution, and metabolism of numerous endogenous and exogenous substances (7, 8). Previous studies have established an association between serum albumin levels at the time of hospital admission in sepsis patients and outcomes such as mortality and renal function (9–11). However, these studies are limited by factors including varied time points for albumin measurement and individual differences in treatment responses, which compromise the accuracy of prognostic assessments (12, 13). To gain a more comprehensive understanding of the impact of albumin levels, dynamic data collection throughout the disease course is necessary (14–17). Although some research suggests that albumin supplementation may be beneficial for vascular function recovery, the findings are inconsistent, and some trials have not confirmed its therapeutic advantage (18, 19). Moreover, given the complexity of sepsis patients, the results of current randomized controlled trials remain controversial and do not fully reflect the specific effects of albumin treatment (20, 21).

Group-based Trajectory Modeling (GBTM) is an individual-focused statistical approach designed to explore the dynamics among individuals by identifying distinct clusters that exhibit similar patterns over time, based on their response behaviors. This method of trajectory modeling enables researchers to more accurately delineate and comprehend the heterogeneity and similarities within and across individuals, as well as to map out patterns of disease progression or recovery over specified periods (22, 23). Applying group trajectory models in the study of sepsis allows for a robust analysis of patient diversity, pinpointing specific patient subgroups that require more intensive scrutiny. This form of modeling can help in stratifying patients according to the progression patterns that align with the most favorable prognoses, thereby facilitating more targeted and effective clinical interventions. Consequently, investigating the variations in serum albumin levels among ICU sepsis patients through trajectory modeling becomes a pivotal step. Such research offers vital insights into the correlations between these fluctuations and potential adverse outcomes, enhancing our capability to predict and improve patient trajectories in sepsis care.

In response to the recognized need for a deeper understanding of serum albumin level dynamics in sepsis patients, we have embarked on a retrospective study leveraging group trajectory models. Our goal is to uncover the association between the trajectories of serum albumin levels and the occurrence of adverse outcomes among sepsis patients. Through this methodology, our objective is to offer enhanced data support for the personalized treatment of sepsis patients, thereby furnishing clearer guidance for tailoring therapeutic approaches to individual needs. This endeavor is pivotal in advancing the precision of sepsis management, ensuring treatments are more effectively aligned with patient-specific conditions and outcomes.

## Materials and methods

### Participants

This retrospective cohort study included patients admitted to the ICU at West China Hospital of Sichuan University from 2015 to 2022. The inclusion criteria comprised patients meeting the Sepsis 3.0 criteria, with exclusion criteria set for individuals younger than 18, patients with ICU stays of less than 48 h or in excess of 100 days, and patients with a history of cancer diagnosis (1). To construct trajectory models, participants with fewer than three albumin tests within the first 7 days post-admission were excluded, ultimately incorporating 1950 participants into the analysis. This study received approval from the Ethics Committee of West China Hospital, Sichuan University (WCH 2023–2333), and was conducted in accordance with the ethical standards of the Declaration of Helsinki. Given its retrospective nature, informed consent was not required. The reporting of this study adheres to the guidelines of the Strengthening the Reporting of Observational Studies in Epidemiology (STROBE) statement (24).

### Independent variables and other covariates

The unit of albumin is g/L, and the covariates considered (Supplementary Table S1 for the selection strategy of covariates) include age, gender, body mass index (BMI; in kg/m2), whether smoking and drinking; vital signs: Body temperature, heart rate, respiratory rate, systolic blood pressure, diastolic blood pressure; disease severity score: Acute Physiology III score (APS-III), Sequential Organ Failure Assessment score (SOFA), Acute Physiology and Chronic Health Evaluation II score (APACHE-II score); Comorbidities: whether there are cardiovascular disease (CVD), hypertension, liver disease, digestive system disease, diabetes, chronic kidney disease, chronic lung disease (Supplementary Table S2 for diagnosis codes). The clinical test indicators include hemoglobin (HB), white blood cells (WBC), platelets (PLT), aspartate aminotransferase (AST), alanine aminotransferase (ALT), direct bilirubin (DBIL), indirect bilirubin (IBIL), creatinine, estimated glomerular filtration rate (eGFR), activated partial thromboplastin time (APTT), prothrombin time (PT), fibrinogen (Fib), C-reactive protein (CRP), procalcitonin (PCT), interleukin-6 (IL-6), partial pressure of oxygen in arterial blood (PaO2), partial pressure of carbon dioxide in arterial blood (PaCO2), residual base (BE), lactic acid. The multiple imputation of chained equations (MICE) technology in R language was used to process missing data. The missing data situation is shown in Supplementary Table S3. Different methods were applied to impute different types of covariates, and 10 data sets were generated, the odds ratio and variance–covariance matrix are estimated separately for each data set. Finally, the 10 data sets were combined into a comprehensive data set following Rubin’s rules to fill in missing data (25).

### Primary and secondary outcomes

This investigation delineated its primary endpoints as 28-day mortality and all-cause mortality during hospitalization. Secondary endpoints encompassed the duration of ICU stay, the occurrence of acute kidney injury (AKI), instances of fluid overload (FO), and the utilization of mechanical ventilation. AKI was specified per the Kidney Disease: Improving Global Outcomes (KDIGO) consensus guidelines as either an increase in serum creatinine by at least 0.3 mg/dL from the baseline within a 48 h window, or a 50% rise relative to baseline within a 7-day timeframe (26). To calculate the daily fluid balance, the total volume of fluid intake—including oral and intravenous sources—was subtracted from the total volume of fluid output, which accounted for urinary output, losses through excreta, and dialysis ultrafiltrate, among other factors. The assessment of FO utilized the following formula: (cumulative fluid balance at the time of evaluation divided by the patient’s baseline weight) multiplied by 100, yielding a percentage. A FO percentage exceeding 10% was the threshold for defining fluid overload (27).

### Group-based trajectory model

In the study, GBTM was employed to examine the variations in serum albumin levels from day 1 to day 7 among participants. GBTM was a semiparametric model tailored for analyzing longitudinal data, operates under the assumption that the population is comprised of discrete, identifiable subgroups. This methodology facilitates the identification of distinct categories within the population, each characterized by homogenous trajectory profiles regarding their albumin levels (22, 23). GBTM categorized albumin trajectories into one to five subgroups, to best represent the changes over the specified period. Determination of the ideal number of trajectory groupings was informed by a combination of criteria, including: (i) the Bayesian Information Criterion (BIC), aiming for values approaching zero to indicate a better model fit; (ii) ensuring each trajectory group comprised more than 5% of the study’s participants, to maintain both relevance and statistical significance; and (iii) the discernibility of visually distinct trajectory paths, confirming the practical distinction among groups. Model fit was rigorously evaluated using two primary metrics: the BIC and the average posterior probability (AvePP) of group membership. A close-to-zero BIC value and an AvePP exceeding 0.7 were indicative of a robust model fit, implying that the identified trajectories are both statistically significant and meaningful in representing the underlying patterns of albumin level changes within the population studied.

### Statistical analysis

In assessing the statistical significance of differences between groups for continuous variables, we utilized the Student’s *t*-test for normally distributed data and the Wilcoxon-Mann–Whitney rank sum test for non-normally distributed data. Categorical variables have been summarized as frequencies and percentages, with the chi-square test applied to evaluate statistical significance among the different albumin trajectory groups. To address issues of missing data within our covariates, we employed a variety of imputation techniques, thereby ensuring the integrity of our subsequent analyses. Following the imputation process, we conducted Kaplan–Meier survival analysis to investigate the prognostic significance of the albumin trajectory groups on 28-day mortality in the ICU setting. This method allowed us to estimate and illustrate the probability of survival over the 28-day period post-ICU admission for the different albumin trajectory groups.

Causal directed acyclic graphs (DAGs) are directed and acyclic graphs that can show the direction of hypothesized causal effects (28). We used DAG to display the variables that may affect the effect of albumin trajectory group on outcome (Supplementary Figure S1). These variables were generated from the covariates we included, including age, gender, BMI, transfer source, vital signs, disease severity score, comorbidities, clinical test indicators, etc. Readers are reminded that these variables in the graph need to be controlled to minimize bias. We used Cox proportional hazards models to examine the relationship between albumin group and outcomes in patients with sepsis, with results reported as hazard ratios (HRs) and 95% confidence intervals (CIs). Two models were conducted, one is a unadjusted rough model, the second is a fully adjusted model (adjusted for variables from DAG).

We performed multiple subgroup analyses to explore potential influencing factors of the relationship between albumin group and outcomes in patients with sepsis, including sex (male vs. female), age (< 65 years vs. ≥ 65 years), comorbidities (yes vs. no), and albumin infusion, etc. We included an interaction term between stratification covariates and albumin group in the model to test for potential effect modification in the fully adjusted model.

All tests were two-sided with a significance level of 0.05. We use Stata software (v17.0; Stata Corporation, College Station, TX, United States), R (v4.2.1; http://www.R-project.org), and Python (v3.7.3; Python Software Foundation) for statistical analysis.

## Results

### Albumin trajectories and baseline characteristics

**Figure 1:**
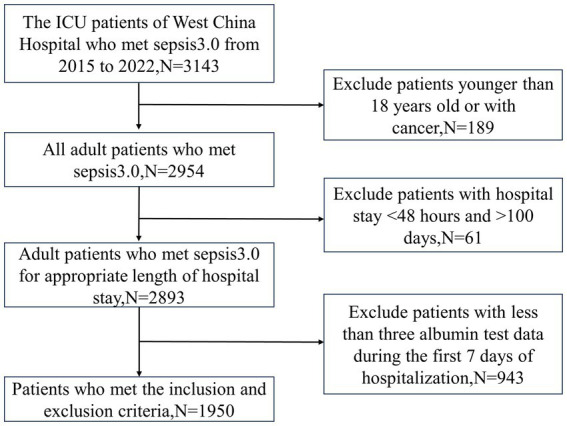
Flowchart of patient selection process.

**Figure 2:**
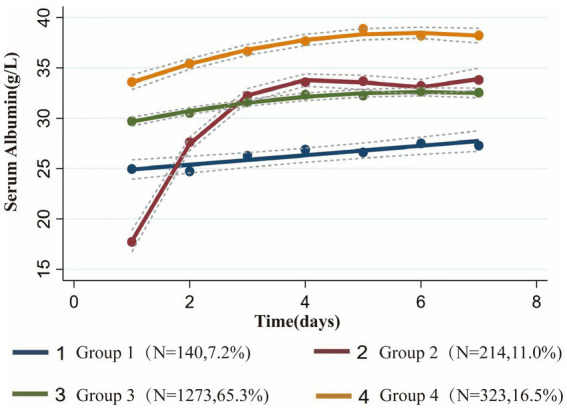
Group-based trajectory modeling of serum albumin levels during the first 7 days after admission to the ICU.

In this study, we recruited 1,950 patients who met the inclusion and exclusion criteria (Figure 1). Based on the BIC and AvePP results (Supplementary Table S4), the albumin trajectory could be divided into four groups; however, one group with an AvePP less than 70% was ultimately excluded, leading us to categorize the albumin trajectories into four distinct groups: Group1 (G1, Stable Low-Level), where albumin remains stable at a low level; Group 2 (G2, Persistent Increase from Low to High Level), where albumin rapidly increases from a low to a high level over time; Group 3 (G3, Stable Mid-Level), where albumin maintains stability at a mid-level; Group 4 (G4, Stable High-Level), where albumin consistently remains stable at a high level (Figure 2). The trend analysis for these four groups is presented in Supplementary Table S5.

Comparing the demographic data, vital signs, laboratory events, and comorbidities among the four groups, significant differences were observed between the clusters for most variables. Notably, the Severity score was the highest for the G2, Persistent Increase from Low to High Level group, including SOFA score/APACHE-II score and APS-III score; the albumin level for this group was also the lowest, at 17.4 ± 4.7 g/L (Table 1).

**Table 1:** Baseline characteristics of the sepsis patients with different serum albumin trajectory groups at admission.

	Overall (*n* = 1,950)	G1 (*n* = 140)	G2 (*n* = 214)	G3 (*n* = 1,273)	G4 (*n* = 323)	*p*
Demographic data						
Age (years)^b^	56.9 ± 17.4	57.8 ± 15.3	54.2 ± 17.1	58.0 ± 17.2	53.7 ± 18.8	<0.001
Gender (*n* %)^c^						0.044
Female	650 (33.3%)	46 (32.9%)	86 (40.2%)	400 (31.4%)	118 (36.5%)	
Male	1,300 (66.7%)	94 (67.1%)	128 (59.8%)	873 (68.6%)	205 (63.5%)	
Smoke (*n* %)^c^	750 (38.5%)	56 (40.0%)	65 (30.4%)	499 (39.2%)	130 (40.3%)	0.079
Drink (*n* %)^c^	512 (26.3%)	44 (31.4%)	45 (21.0%)	349 (27.4%)	74 (22.9%)	0.052
Physical examination findings						
BMI (kg/m^2^)^b^	23.3 ± 3.6	22.9 ± 3.1	22.5 ± 3.4	23.4 ± 3.6	23.5 ± 3.8	0.004
Body temperature (T°C)^b^	37.0 ± 0.8	37.1 ± 0.8	37.0 ± 0.8	37.0 ± 0.8	37.0 ± 0.8	0.006
Heart rate (beats/min)^b^	97 ± 21	98 ± 18	101 ± 21	97 ± 20	93 ± 18	<0.001
Respiratory rate (beats/min)^b^	19 ± 4	19 ± 4	18 ± 4	19 ± 4	18 ± 4	<0.001
SBP (mmHg)^b^	124 ± 18	124 ± 18	119 ± 16	125 ± 17	125 ± 19	<0.001
DBP (mmHg)^b^	70 ± 12	70 ± 13	68 ± 11	70 ± 12	71 ± 13	0.017
Disease Severity score						
SOFA score^b^	10 [8, 13]	11 [9, 14]	12 [10, 14]	10 [8, 13]	9 [7, 12]	<0.001
APACHE-II score^b^	25 [21, 28]	25 [22, 29]	26 [22, 29]	25 [21, 28]	24 [20, 28]	<0.001
APS-III score^b^	21 [18, 24]	21 [18, 25]	23 [19, 26]	21 [18, 24]	20 [18, 24]	<0.001
Main infection site						
Respiratory	427 (21.9%)	21 (15.0%)	45 (21.0%)	270 (21.2%)	88 (27.2%)	0.021
Abdominal	1,113 (57.1%)	87 (62.1%)	129 (60.3%)	729 (57.3%)	168 (52.0%)	0.450
Genitourinary	214 (11.0%)	13 (9.3%)	19 (8.9%)	146 (11.5%)	36 (11.1%)	0.945
Skin/soft tissue	196 (10.0%)	19 (13.6%)	21 (9.8%)	125 (9.8%)	31 (9.6%)	0.912
Comorbidities (*n* %)						
Cardiovascular disease^c^	191 (9.8%)	14 (10.0%)	5 (2.3%)	126 (9.9%)	46 (14.2%)	<0.001
Hypertension^c^	649 (33.3%)	32 (22.9%)	36 (16.8%)	472 (37.1%)	109 (33.8%)	<0.001
Liver disease^c^	236 (12.1%)	20 (14.3%)	24 (11.2%)	158 (12.4%)	34 (10.5%)	0.647
Digestive disease^c^	629 (32.3%)	54 (38.6%)	80 (37.4%)	412 (32.4%)	83 (25.7%)	0.009
Diabetes^c^	440 (22.6%)	37 (26.4%)	37 (17.3%)	300 (23.6%)	66 (20.4%)	0.103
Kidney disease^d^	73 (3.7%)	3 (2.1%)	3 (1.4%)	56 (4.4%)	11 (3.4%)	0.123
Pulmonary disease^c^	153 (7.9%)	8 (5.7%)	9 (4.2%)	111 (8.7%)	25 (7.7%)	0.105
Laboratory results						
HB (g/L)^a^	92.7 ± 25.5	90.1 ± 20.6	89.5 ± 26.6	92.1 ± 25.0	98.2 ± 27.5	<0.001
WBC (*10^9^/L)^b^	10.8 [7.5, 15.1]	11.60[7.0, 16.5]	10.1 [5.9, 14.2]	10.8 [7.6, 14.9]	11.3 [8.3, 15.2]	0.026
PLT (*10^9^/L)^b^	125.0 [71.0, 192.0]	117.50 [62.8, 163.8]	104.0[47.3, 172.5]	126.0 [74.0, 196.0]	136.0[76.0, 203.0]	<0.001
Albumin (g/L)^a^	29.0 ± 6.5	24.9 ± 4.6	17.4 ± 4.7	29.9 ± 4.2	34.4 ± 5.7	<0.001
AST (U/L)^b^	42.0 [24.0, 85.8]	42.5 [24.0, 88.5]	51.5 [28.0, 124.5]	42.0 [25.0, 82.0]	36.0 [22.0, 74.5]	0.003
ALT (U/L)^b^	28.0 [16.0, 62.0]	27.0 [15.0, 53.0]	28.0 [15.0, 57.8]	29.0 [16.0, 62.0]	27.0 [16.0, 70.0]	0.971
DBIL (μmol/L)^b^	8.5 [4.8, 18.7]	12.0 [6.6, 28.0]	11.5 [6.3, 21.0]	8.3 [4.7, 18.6]	6.3 [3.9, 13.5]	<0.001
IBIL (μmol/L)^b^	5.8 [3.4, 10.2]	5.1 [3.2, 7.9]	4.7 [2.4, 7.5]	5.9 [3.4, 10.3]	7.4 [4.4, 12.0]	<0.001
Serum creatinine (μmol/L)^b^	82.0 [57.0, 146.8]	91.0 [57.8, 150.7]	85.7 [62.3, 131.8]	85.0 [56.0, 158.0]	75.0 [55.0, 114.0]	0.05
eGFR (ml/min/1.73 m^2^)^a^	76.0 ± 39.1	71.9 ± 39.6	75.7 ± 35.9	74.9 ± 39.8	82.0 ± 38.0	0.014
APTT (s)^a^	40.3 ± 20.1	41.6 ± 13.4	63.2 ± 32.9	37.5 ± 15.6	35.7 ± 16.6	<0.001
PT (s)^a^	15.0 ± 5.9	15.7 ± 5.7	17.6 ± 5.4	14.8 ± 6.3	13.7 ± 3.8	<0.001
Fib (g/L)^a^	3.7 ± 1.9	3.7 ± 2.0	2.6 ± 1.7	3.9 ± 1.9	3.6 ± 1.9	0.001
CRP (mg/L)^b^	107.4 [64.2, 169.0]	145.8 [82.4, 212.8]	118.2 [78.6, 172.8]	111.0 [68.5, 171.0]	77.8 [33.5, 128.0]	<0.001
PCT (ng/ml)^b^	1.7 [0.5, 8.7]	4.6 [1.3, 15.3]	8.4 [1.8, 29.2]	1.5 [0.4, 7.0]	0.8 [0.2, 3.2]	<0.001
IL-6 (pg/ml)^b^	135.1 [52.4, 378.4]	315.9 [123.2, 698.7]	515.9 [169.1, 944.6]	125.8 [52.0, 311.8]	74.8 [32.6, 193.7]	<0.001
PaO2 (mmHg)^a^	111.8 ± 41.9	104.8 ± 36.6	117.0 ± 46.7	110.6 ± 42.0	116.5 ± 39.6	0.081
PaCO2 (mmHg)^a^	38.7 ± 9.0	36.6 ± 9.0	36.5 ± 7.8	39.0 ± 9.1	39.5 ± 8.7	<0.001
ABE (mmol/L)^a^	−1.2 ± 4.9	−2.5 ± 4.8	−3.6 ± 4.2	−0.9 ± 4.9	−0.3 ± 4.7	<0.001
SBE (mmol/L)^a^	−1.4 ± 5.5	−2.8 ± 5.2	−4.0 ± 4.7	−1.0 ± 5.5	−0.3 ± 5.3	<0.001
Lactic acid (mmol/L)^b^	1.9 [1.4, 3.1]	2.2 [1.6, 3.5]	3.3 [1.9, 5.7]	1.8 [1.4, 2.7]	1.9 [1.4, 3.0]	<0.001

Group 1 (G1, Stable Low-Level); Group 2 (G2, Persistent Increase from Low to High Level); Group 3 (G3, Stable Mid-Level); Group 4 (G4, Stable High-Level). Presented are the comparison of demographics, Physical examination findings, comorbidities and Laboratory results between serum albumin trajectory groups 1–4. Data conforming to a normal distribution are presented as mean ± standard deviation, while non-normally distributed data are displayed as median along with interquartile ranges. BMI, body mass index; SBP, systolic blood pressure; DBP, diastolic blood pressure; APS-III score, acute physiology III score; SOFA score, sequential organ failure assessment score; APACHE-II score, acute physiology and chronic health evaluation II score; ICU, intensive care unit; WBC, white blood cell; HB, hemoglobin; PLT, platelet; AST, aspartate aminotransferase; ALT, alanine aminotransferase; DBIL, direct bilirubin; IBIL, indirect bilirubin; eGFR, estimated glomerular filtration rate; APTT, activated partial thromboplastin time; PT, prothrombin time; Fib, fibrinogen; CRP, C-reactive protein; PCT, procalcitonin; IL-6, interleukin-6; PaO2, partial pressure of oxygen in arterial blood; PaCO2, partial pressure of carbon dioxide in arterial blood; ABE, actual base excess; SBE, standard base excess.

a F-test.

b Kruskal-Wallis test.

c Chi-square test.

d Fisher’s precision probability test.

### Association between albumin trajectories and clinical outcomes

**Figure 3:**
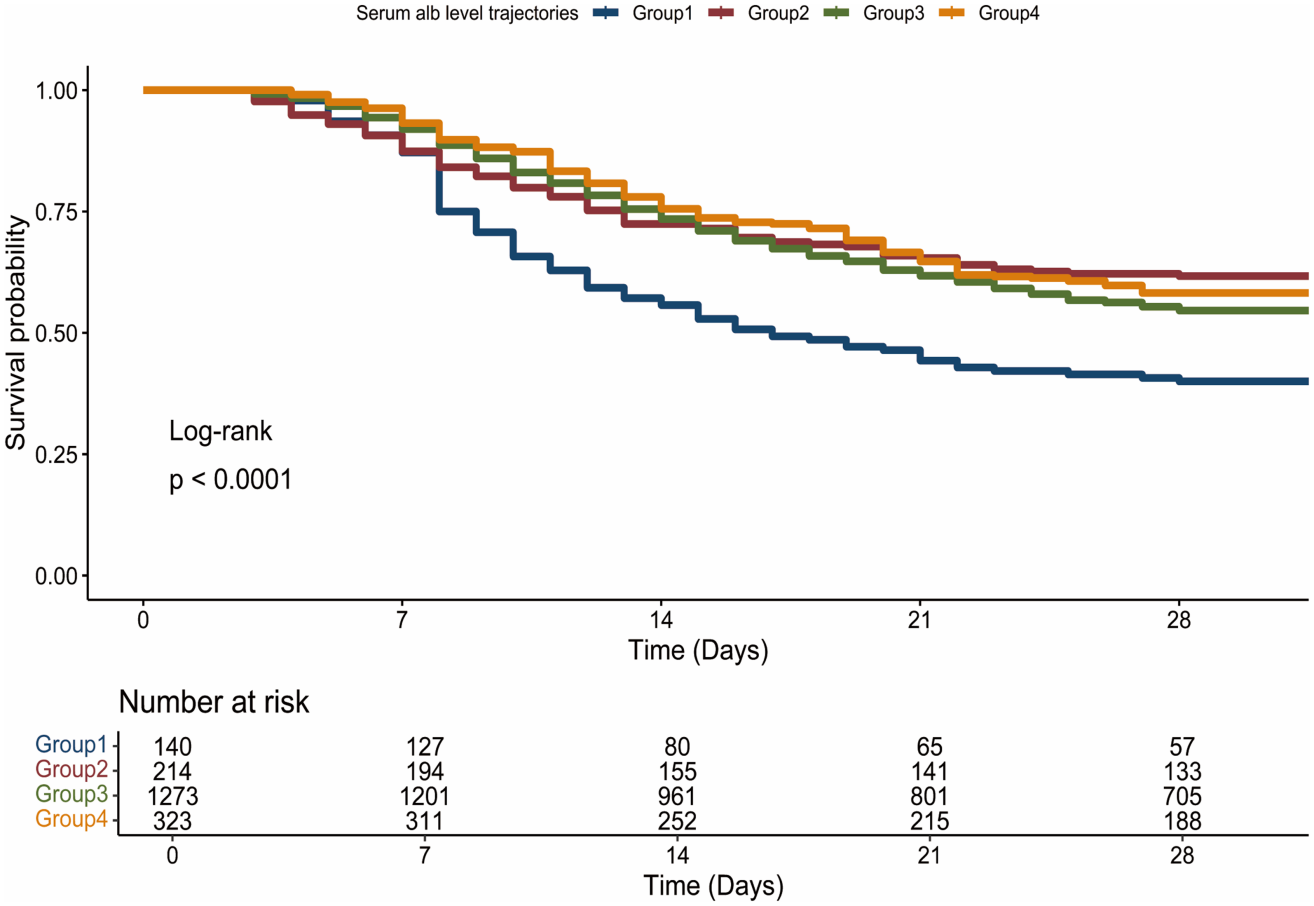
Association between serum albumin trajectories and 28-day Mortality.

The all-cause and 28-day mortality rates were markedly higher in the Stable Low-Level group, characterized by a higher incidence of acute kidney injury (AKI) and fluid accumulation, as well as an increased requirement for mechanical ventilation, particularly noted in G2, Persistent Increase from Low to High Level compared to the other cohorts (Table 2; Supplementary Figure S2). Kaplan–Meier survival analysis uncovered substantial variances in mortality rates across the different patient groups, with G1, Stable Low-Level experiencing the highest mortality, while Groups 2, 3, and 4 displayed intermediate rates of mortality (Figure 3). Group 1’s mortality risk was significantly greater compared to the latter groups, each of which exhibited a marked decrease in the risk of death with *p*-values less than 0.05 (Supplementary Table S6-1). Multivariate Cox regression analysis further corroborated these findings, indicating a considerable reduction in mortality risk for G2, Persistent Increase from Low to High Level with a hazard ratio (HR) of 0.52 (95% CI: 0.38–0.71). Groups with Stable Mid-Level and Stable High-Level albumin also showed a decreased mortality risk, with HRs of 0.61 (95% CI: 0.49–0.77) and 0.55 (95% CI: 0.42–0.72) respectively, and these trends persisted after full adjustment. Nonetheless, no significant differences in the risk for 28-day mortality were found between G2, G3, and G4 groups (Table 3; Supplementary Tables S6-2–S6-4).

**Table 2:** Clinical outcomes of the study patients with different serum albumin trajectory groups.

Outcome	Overall (*n* = 1,950)	G1 (*n* = 140)	G2 (*n* = 214)	G3 (*n* = 1,273)	G4 (*n* = 323)	*p*
Overall hospital mortality	1,089 (55.8%)	96 (68.6%)	102 (47.7%)	729 (57.3%)	162 (50.2%)	<0.001
28-day mortality (*n*, %)	879 (45.1%)	84 (60.0%)	82 (38.3%)	578 (45.4%)	135 (41.8%)	<0.001
Length of ICU stay (days)	14 [9, 24]	11 [8, 20]	11 [8, 18]	15 [9, 25]	14 [9, 23]	<0.001
Acute renal failure (*n*, %)	1,211 (62.1%)	93 (66.4%)	121 (56.5%)	812 (63.8%)	185 (57.3%)	0.034
Ventilation (*n*, %)						
Iv	1830 (93.8%)	121 (86.4%)	209 (97.7%)	1,198 (94.1%)	302 (93.5%)	<0.001
Niv	539 (27.6%)	31 (22.1%)	58 (27.1%)	361 (28.4%)	89 (27.6%)	0.482
Average daily ventilation time (hours per day)	13.1 ± 11.9	11.7 ± 12.3	10.6 ± 9.8	13.8 ± 12.3	12.3 ± 11.0	0.001
Fluid overload (*n*, %)	902 (46.3%)	68 (48.6%)	85 (39.7%)	597 (46.9%)	152 (47.1%)	0.233

Group 1 (G1, Stable Low-Level); Group 2 (G2, Persistent Increase from Low to High Level); Group 3 (G3, Stable Mid-Level); Group 4 (G4, Stable High-Level). The table illustrates the clinical outcomes for different serum albumin trajectory groups. Primary outcomes include overall hospital mortality and 28-day mortality. Secondary outcomes encompass the length of ICU stay, the incidence of acute renal failure during hospitalization, the proportion of Fluid overload, and ventilator usage scenarios. Iv, invasive mechanical ventilation; Niv, noninvasive mechanical ventilation.

**Table 3:** Multivariable Cox regression analysis for different albumin trajectory groups and 28-day mortality.

Alb Group	Model 1		Model 2	
	HR (95%CI)	*p*	HR (95%CI)	*p*
Group 1	1	Reference	1	Reference
Group 2	0.52 (0.38–0.71)	<0.001	0.51 (0.37–0.69)	<0.001
Group 3	0.61 (0.49–0.77)	<0.001	0.64 (0.51–0.81)	<0.001
Group 4	0.55 (0.42–0.72)	<0.001	0.62 (0.47–0.82)	<0.001

Group 1 (G1, Stable Low-Level); Group 2 (G2, Persistent Increase from Low to High Level); Group 3 (G3, Stable Mid-Level); Group 4 (G4, Stable High-Level). Model 1: Unadjusted rough model. Model 2: Fully adjusted model (adjusted for variables from DAG).

### Robust analysis

**Figure 4:**
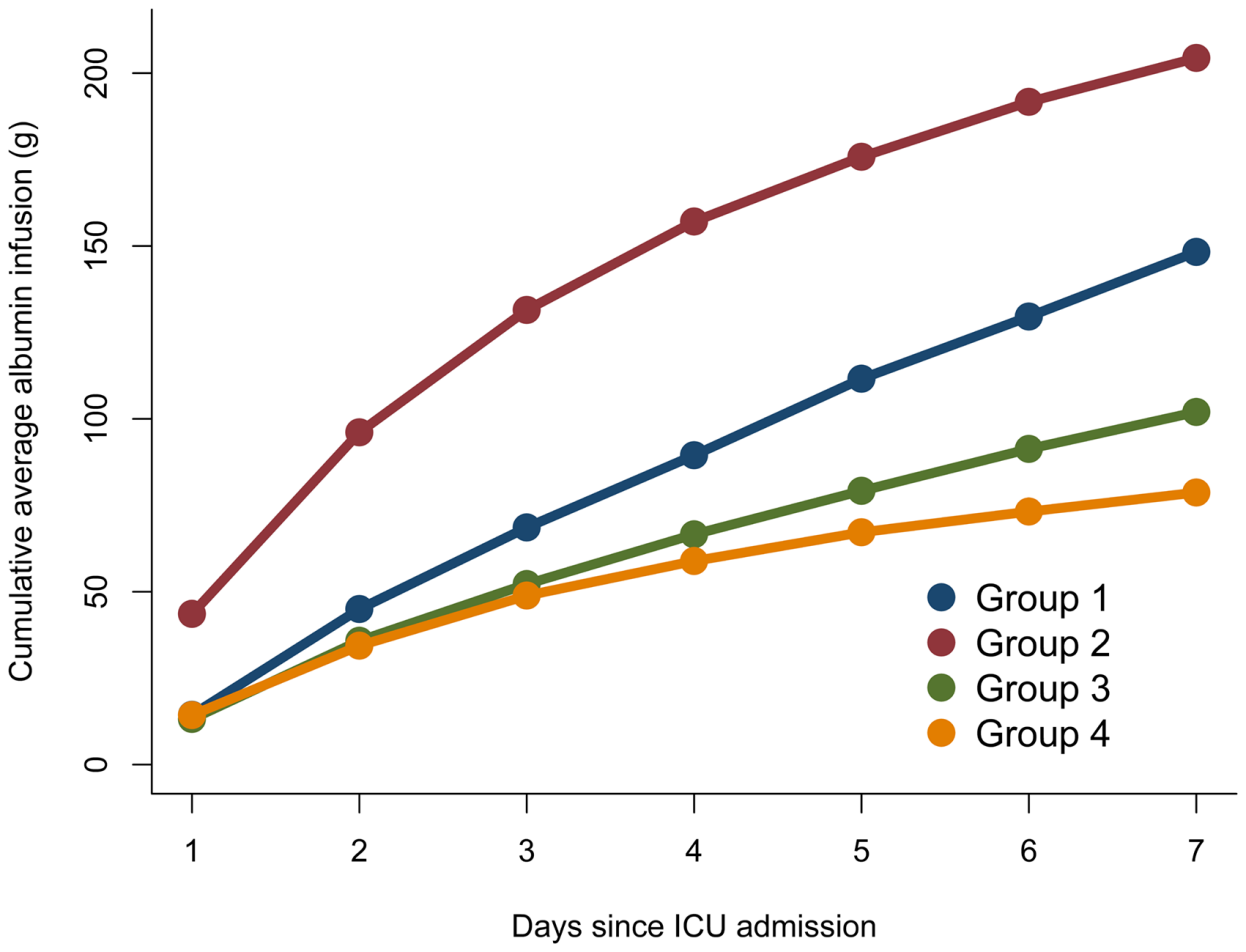
Cumulative average albumin infusion compared between different serum albumin trajectory sub-groups.

**Figure 5:**
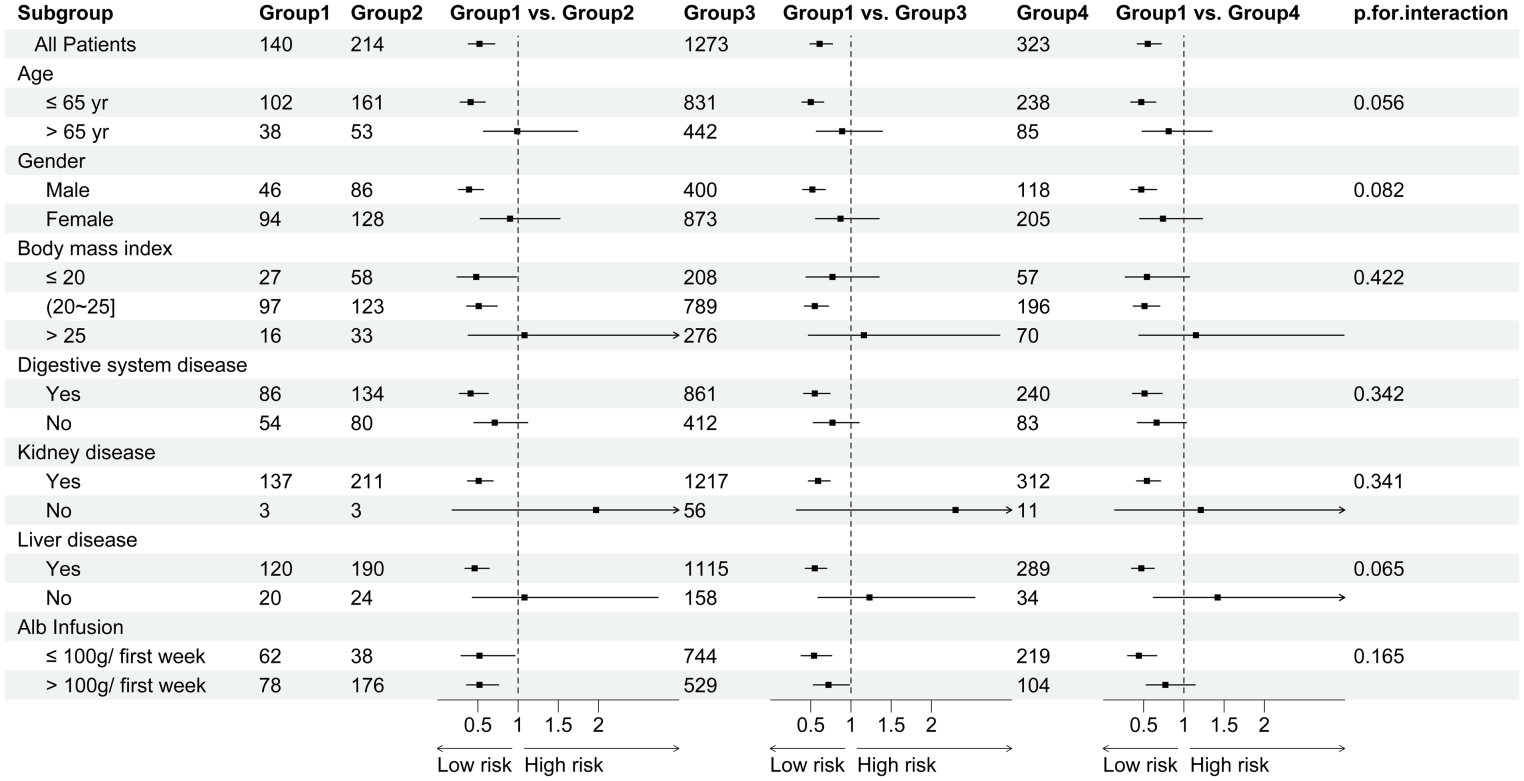
The subgroup analysis based sub-groups and ICU 28-day mortality.

To elucidate the specific impact of albumin infusion on its trajectory alterations, we have meticulously detailed the relevant information regarding albumin infusion in Table 4 and provided a visual representation in Figure 4. Subgroup analyses were rigorously performed for a multitude of demographic and clinical parameters, including age, gender, comorbidities, and albumin infusion as depicted in Figure 5, encompassing a broad spectrum of characteristics such as age, gender, Body Mass Index (BMI), liver disease, diseases of the digestive system, kidney disease, and albumin infusion. Within these well-defined subgroups, the association between dynamic albumin categories and corresponding mortality rates did not exhibit statistical significance (*p* > 0.05), indicating a consistent relationship across different patient demographics. This outcome suggests that the prognostic relevance of dynamic albumin levels on mortality is not affected by these specified factors, thereby maintaining its predictive integrity across varying patient characteristics.

**Table 4:** The amount of albumin used in different albumin trajectory groups.

	Overall (*n* = 1,950)	G1 (*n* = 140)	G2 (*n* = 214)	G3 (*n* = 1,273)	G4 (*n* = 323)	*p*
Albumin utilization rate	75.9%	77.1%	93.9%	76.4%	61.6%	<0.001
Total albumin use per patient (g)	112.9	148.3	204.4	102	78.7	<0.001
Total albumin use per person per day (g)	16.1	21.2	29.2	14.6	11.2	<0.001
Day1	16.8 (16.8)	14.4 (14.4)	43.6 (43.6)	13.2 (13.2)	14.3 (14.3)	<0.001
Day2	26.1 (42.9)	30.6 (45)	52.5 (96.1)	22.6 (35.8)	20.1 (34.4)	<0.001
Day3	18.7 (61.6)	23.6 (68.6)	35.4 (131.5)	16.4 (52.2)	14.5 (48.9)	<0.001
Day4	15.4 (77)	20.9 (89.5)	25.6 (157.1)	14.4 (66.6)	10 (58.9)	<0.001
Day5	13.3 (90.3)	22.1 (111.6)	18.7 (175.8)	12.6 (79.2)	8.3 (67.2)	<0.001
Day6	11.9 (102.2)	18 (129.6)	15.9 (191.7)	12.1 (91.3)	6 (73.2)	<0.001
Day7	10.7 (112.9)	18.7 (148.3)	12.7 (204.4)	10.7 (102)	5.5 (78.7)	<0.001

Group 1 (G1, Stable Low-Level); Group 2 (G2, Persistent Increase from Low to High Level); Group 3 (G3, Stable Mid-Level); Group 4 (G4, Stable High-Level). The table displays the albumin infusion practices within the first 7 days in the ICU for different serum albumin trajectory groups, including the overall utilization rate and the total amount used. Daily usage is presented for Day 1 through Day 7, with the cumulative total amount used at each time point indicated in parentheses.

## Discussion

This study utilized GBTM to investigate the diverse evolving trajectories of serum albumin levels in sepsis patients. Notably, this research pioneers the examination of the association between dynamic changes in serum albumin levels among adult sepsis patients and their adverse clinical outcomes. Our findings reveal several key insights: initially, we identified four unique serum albumin trajectory groups (G1, Stable Low-Level; G2, Persistent Increase from Low to High Level; G3, Stable Mid-Level; G4, Stable High-Level), and assessed their correlation with poor prognostic outcomes in the ICU setting. We observed that the trend of serum albumin level changes in the 7 days preceding ICU admission was highly indicative of patient prognosis. The incidence of adverse outcomes progressively increased with decreasing serum albumin levels across the three groups with stable states (G1, G3, G4). The G2, Persistent Increase from Low to High Level exhibited a significantly more favorable prognosis than the persistently low group (G1), and patients in this rising trajectory group were also less likely to experience adverse outcomes compared to those maintaining consistently moderate (G3) and persistently high (G4) levels.

The four distinct serum albumin trajectories uncovered in this study highlight the heterogeneity among sepsis patients, suggesting that differing prognoses are associated with varying serum albumin trends. Our research indicates that for the majority of sepsis patients, serum albumin levels remained stable during the initial 7 days. Among these patients with steady albumin trajectories, G1, Stable Low-Level, characterized by persistently low levels, exhibited serum albumin fluctuations between 25 g/L and 27 g/L; G3, Stable Mid-Level, with consistently moderate levels, fluctuated between 30 g/L and 33 g/L; and G4, Stable High-Level, maintaining elevated levels, varied between 34 g/L and 38 g/L. The prognosis for these patients worsened as serum albumin levels decreased, corroborating previous findings that for every 10 g/L reduction in serum albumin concentration, an increase in the incidence of sepsis, mortality rates, and length of hospital stay was observed (11, 16, 29). We identified a critical threshold for serum albumin levels in sepsis patients at 30 g/L, below which the likelihood of mortality and acute kidney injury significantly increases. Compared to the serum albumin trajectories observed in previous RCTs among sepsis patients, which predominantly focused on the groups with serum albumin levels around 30 g/L and 25 g/L, the discrepancies in outcomes could be attributed to several factors: 1. Stringent inclusion criteria may reduce the observed heterogeneity inherent among sepsis patients; 2. Variations in initial serum albumin levels among patients in different trials could lead to divergent outcomes; 3. Trial-directed interventions, such as artificial albumin infusions, may not accurately reflect real clinical scenarios, potentially skewing the results. Investigating the natural trajectories of serum albumin changes in patients without prior artificial grouping or interventions indeed offers a more accurate reflection of real clinical scenarios, leading to stronger and more relevant conclusions. Our study also explores the trajectories of serum albumin levels in sepsis patients above 30 g/L, a patient subset often overlooked in past research, resulting in a lack of guidelines for the use of albumin therapy in this group. Our analysis suggests that in sepsis patients with albumin levels exceeding 30 g/L, further elevation of albumin levels potentially continues to ameliorate the risks of mortality and acute kidney injury, albeit with a relatively marginal benefit that did not reach statistical significance in our study (G3 vs. G4). This finding underscores the importance of maintaining serum albumin levels at 30 g/L as the primary treatment goal for sepsis patients. Once this target is reached, the infusion of albumin could be gradually reduced or ceased to prevent wastage and avoid excessive healthcare costs (30–32).

Our study has revealed that serum albumin levels in patients suffering from sepsis do not invariably remain stable; a minority of patients exhibit rapid fluctuations (G2). Among these patients experiencing a progressive rise in serum albumin levels, the serum albumin concentration upon admission to the ICU was found to be the lowest, accompanied by the administration of a significantly higher volume of albumin during the initial 7 days of ICU stay. Existing literature suggests that hypoalbuminemia (commonly defined as a serum albumin concentration ≤ 30 g/L) constitutes a prognostic indicator of adverse outcomes in critically ill patients. Consequently, one might anticipate that this cohort would exhibit the most unfavorable prognosis (16, 33–35). However, our analysis of dynamic trajectories reveals that hypoalbuminemia at the point of ICU admission does not directly correlate with the prognosis for all patients. Instead, attention should be directed toward the changes in albumin levels throughout the treatment period. Should the low levels of serum albumin be promptly corrected, patients often demonstrate the most favorable clinical outcomes (G1 vs. G2). This finding corroborates previous research that emphasizes the immediate rectification of hypoalbuminemia in sepsis patients. It further elucidates the strong association between the patterns of serum albumin level changes post-treatment in sepsis patients and their prognoses, offering crucial insights for clinicians in devising targeted treatment strategies (21, 30).

Our study has revealed significant disparities in the duration of ICU stay, the length of mechanical ventilation, and the incidence of acute kidney injury and fluid accumulation among patient cohorts with divergent serum albumin trajectories. The cohort characterized by persistently low albumin levels exhibited the highest likelihood of developing acute kidney injury and fluid accumulation, with their shorter ICU stay potentially attributable to an increased mortality rate within a brief period. Conversely, the group with a consistent rise from low albumin levels demonstrated the lowest probability of such complications. This phenomenon can be elucidated through existing theoretical frameworks: the endothelial glycocalyx, a delicate structure that lines the surface of vascular endothelium, is composed of proteoglycans, glycosaminoglycan side chains, and plasma proteins such as albumin and antithrombin (12, 36, 37). During sepsis, the endothelial glycocalyx is one of the earliest and most critical sites of damage. The desquamation of this vascular glycocalyx leads to capillary leakiness and a loss of vascular reactivity, culminating in edema and fluid retention. Adhesion molecules, such as E-selectin and intercellular adhesion molecule 1, become exposed on the denuded endothelium, promoting the recruitment of leukocytes and platelets. This cascade contributes to thrombus formation and, in concert with extensive fibrin deposition, precipitates circulatory dysfunction. Such alterations in blood flow and impaired oxygen delivery can quickly lead to organ failure, with kidneys being among the first affected due to their significant blood supply (38–40). The intimate relationship between the vascular endothelial glycocalyx and albumin offers us valuable insights into the elucidation of various pathophysiological states commonly observed in ICU patients, including circulatory dysfunction, diminished vascular reactivity, fluid accumulation, acute kidney injury, acute lung injury, and pulmonary edema.

It is imperative to acknowledge that in patients with sepsis in the ICU, the fluctuations in serum albumin levels are inextricably linked to the volume of exogenous albumin administered (41). To delve deeper into the relationship between albumin administration and the variation in serum albumin levels, we charted the volume of albumin infusions given to different patient groups over the first 7 days, observing the alterations in serum albumin levels from the perspective of albumin infusion volume. Our findings reveal a significant correlation between the volume of administered albumin and the patients’ albumin levels at the time of admission. Specifically, the lower the albumin level at admission, the greater the volume of albumin received in the initial phase of treatment. We postulate that the differences in albumin administered during treatment are not solely responsible for the distinct patient trajectories observed. For instance, G1 and G2 both commenced with low albumin levels and received substantial albumin infusions each day of treatment, yet their trajectories diverged markedly. Conversely, G3 and G4 had higher albumin levels at admission and minimal variance in their albumin infusion volumes during treatment, yet still maintained differing stable trajectories. We conjecture that the disparate patterns of serum albumin trajectory are greatly influenced by the patients’ individual clinical conditions. G2’s patients had more severe conditions at admission, as reflected by higher SOFA scores and more pronounced inflammatory responses. The factors contributing to their favorable therapeutic response may include a higher proportion of female patients, fewer diabetic complications, and younger age, which could confer more resilient vascular conditions allowing for more effective restoration of endothelial function post albumin therapy (42, 43). On the other hand, among the patients in G4, who received the minimal volume of albumin infusions, their younger age and the least severe inflammatory responses at the time of admission could be factors enabling them to maintain comparatively higher levels of serum albumin (39, 44). For this group of patients, further prospective studies are indeed necessary to clarify the situation. The results of this part of the research suggest that albumin infusions play a significant role in the management of ICU patients with sepsis, but the therapy is merely one means of controlling the variation of albumin levels within the patient’s body. In the future, it seems that greater attention should be directed toward the trends in albumin trajectory changes that result from all influencing factors in patients. This holistic view can provide a more comprehensive understanding of how albumin levels are affected by various clinical interventions and the natural course of the patient’s illness. By taking into account the myriad of factors that can influence albumin levels, such as nutritional status, fluid balance, liver function, and the severity of illness, healthcare professionals can tailor their therapeutic approaches to better suit individual patient needs and potentially improve outcomes.

This study boasts several strengths: heretofore, no research has delineated the relationship between the spontaneous trajectory of albumin levels in ICU patients with sepsis and the risk of mortality, AKI, and fluid accumulation, among other adverse prognostic outcomes. Additionally, this investigation addresses a knowledge gap regarding the prognostic impact of albumin level fluctuations in patients with sepsis when albumin exceeds 30 g/L, thus providing a data-driven foundation for future therapeutic interventions. Nonetheless, the study is not without its limitations. Despite employing a variety of statistical methodologies to examine the association between albumin trajectory groups and unfavorable ICU outcomes, one cannot wholly negate the potential confounding effects of covariates present within the research; To delve deeper into this issue, we recommend using marginal structural modeling for analysis in future studies and conducting more prospective and rigorously designed research (45). Moreover, given our status as a single-center database with a patient cohort admitted to our ICU that generally presents with more severe conditions, our study’s scope is inherently limited. Additionally, the influence of other treatments, such as nutritional support, on patient albumin trajectories remains indeterminate. Lastly, owing to the retrospective observational nature of this study, the relationship between albumin trajectory groups and ICU mortality rates can merely be posited, necessitating further prospective research to establish causality.

## Conclusion

In our retrospective cohort study, four distinct serum albumin trajectory groups were identified in sepsis patients. Patients with a sustained low level of albumin within the first 7 days of ICU admission exhibited the highest likelihood of adverse outcomes, including mortality. Conversely, patients with a sustained increase in albumin levels demonstrated the best prognosis. Among sepsis patients with stable albumin levels above 30 g/L, patients in different trajectory groups exhibited similar outcomes. These four groups can assist in guiding clinical management, enabling early identification of patients with poor prognosis, and providing valuable insights for clinical practice.

## Data availability statement

The data analyzed in this study is subject to the following licenses/restrictions: data will be made available on request. Requests to access these datasets should be directed to heymax633@foxmail.com.

## Ethics statement

The studies involving humans were approved by the Ethics Committee of the West China Hospital provided ethics approval of this work (WCH 2023–2333). The studies were conducted in accordance with the local legislation and institutional requirements. The human samples used in this study were acquired from primarily isolated as part of your previous study for which ethical approval was obtained. Written informed consent for participation was not required from the participants or the participants’ legal guardians/next of kin in accordance with the national legislation and institutional requirements.

## Author contributions

XT: Writing – original draft. YZ: Writing – original draft. TS: Writing – original draft. RZ: Data curation, Writing – review & editing. JL: Resources, Writing – review & editing. JS: Validation, Visualization, Writing – review & editing. WY: Project administration, Writing – review & editing.

## Glossary

ABE: Actual base excess
AKI: Acute kidney injury
ALT: Alanine aminotransferase
APACHE-II score: Acute Physiology and Chronic Health Evaluation II score
APS-III score: Acute Physiology III score
APTT: Activated partial thromboplastin time
AST: Aspartate aminotransferase
AvePP: Average posterior probability
BIC: Bayesian Information Criterion
BMI: Body mass index
CI: Confidence interval
CRP: C-reactive protein
CVD: Cardiovascular disease
DAG: Directed acyclic graphs
DBIL: Direct bilirubin
DBP: Diastolic blood pressure;
eGFR: estimated glomerular filtration rate
Fib: Fibrinogen
FO: Fluid overload
GBTM: Group-based Trajectory Modeling
HB: Hemoglobin
HR: Hazard ratio
IBIL: Indirect bilirubin
ICU: Intensive Care Unit
IL-6: Interleukin-6
KDIGO: The Kidney Disease Improving Global Outcomes
MICE: Multiple imputation of chained equations
PaCO2: Partial Pressure of Carbon Dioxide in Arterial Blood
PaO2: Partial pressure of oxygen in arterial blood
PCT: Procalcitonin
PLT: Platelet
PT: Prothrombin time
SBE: Standard base excess
SBP: Systolic blood pressure
SOFA score: Sequential Organ Failure Assessment score
STROBE: Strengthening the Reporting of Observational Studies in Epidemiology
WBC: white blood cell
