# Supplementary material for: Associations between serum albumin level trajectories and clinical outcomes in sepsis patients in ICU: insights from longitudinal group trajectory modeling
Source: Front Nutr. 2024 Jul 19;11:1433544. doi: 10.3389/fnut.2024.1433544 (PMC11294201; doi:10.3389/fnut.2024.1433544)

**Table S1: Selection strategy for variables with multiple measurements**

| Data items | Details |
| --- | --- |
| Age | Record the initial documentation upon admission to the ICU |
| Gender | Record the initial documentation upon admission to the ICU |
| Smoke | Record the initial documentation upon admission to the ICU |
| Drink | Record the initial documentation upon admission to the ICU |
| BMI | Record the initial documentation upon admission to the ICU |
| Body temperature | Record the highest value for 24 hours of ICU admission |
| Heart rate | Record the highest value for 24 hours of ICU admission |
| Respiratory rate | Record the highest value for 24 hours of ICU admission |
| SBP | Record the initial documentation upon admission to the ICU |
| DBP | Record the initial documentation upon admission to the ICU |
| Disease Severity score | |
| SOFA score | Record the highest value for 24 hours of ICU admission |
| APACHE-II score | Record the highest value for 24 hours of ICU admission |
| APS-III score | Record the highest value for 24 hours of ICU admission |
| Comorbidities | |
| Cardiovascular disease | Record the initial documentation upon admission to the ICU |
| Hypertension | Record the initial documentation upon admission to the ICU |
| Liver disease | Record the initial documentation upon admission to the ICU |
| Digestive disease | Record the initial documentation upon admission to the ICU |
| Diabetes | Record the initial documentation upon admission to the ICU |
| Kidney disease | Record the initial documentation upon admission to the ICU |
| Pulmonary disease | Record the initial documentation upon admission to the ICU |
| Biology | |
| HB | Record the lowest value for 24 hours of ICU admission |
| WBC | Record the highest value for 24 hours of ICU admission |
| PLT | Record the lowest value for 24 hours of ICU admission |
| Albumin | Record the lowest value for 24 hours of ICU admission |
| AST | Record the highest value for 24 hours of ICU admission |
| ALT | Record the highest value for 24 hours of ICU admission |
| DBIL | Record the highest value for 24 hours of ICU admission |
| IBIL | Record the highest value for 24 hours of ICU admission |
| Serum creatinine | Record the highest value for 24 hours of ICU admission |
| eGFR | Record the lowest value for 24 hours of ICU admission |
| APTT | Record the highest value for 24 hours of ICU admission |
| PT | Record the highest value for 24 hours of ICU admission |
| Fib | Record the highest value for 24 hours of ICU admission |
| CRP | Record the highest value for 24 hours of ICU admission |
| PCT | Record the highest value for 24 hours of ICU admission |
| IL-6 | Record the highest value for 24 hours of ICU admission |
| PaO2 | Record the lowest value for 24 hours of ICU admission |
| PaCO2 | Record the highest value for 24 hours of ICU admission |
| ABE | Record the highest value for 24 hours of ICU admission |
| SBE | Record the highest value for 24 hours of ICU admission |
| Lactic acid | Record the highest value for 24 hours of ICU admission |

BMI: body mass index; SBP: systolic blood pressure; DBP: diastolic blood pressure; APS-III score: Acute Physiology III score; SOFA score: Sequential Organ Failure Assessment score; APACHE-II score:Acute Physiology and Chronic Health Evaluation II score; ICU: intensive care unit; WBC: white blood cell; HB: hemoglobin; PLT: platelet; AST: aspartate aminotransferase; ALT: alanine aminotransferase; DBIL: direct bilirubin; IBIL: indirect bilirubin; eGFR: estimated glomerular filtration rate; APTT: activated partial thromboplastin time; PT: prothrombin time; Fib: fibrinogen; CRP: C-reactive protein; PCT: procalcitonin; IL-6: interleukin-6; PaO2: partial pressure of oxygen in arterial blood; PaCO2: Partial Pressure of Carbon Dioxide in Arterial Blood; ABE: actual base excess; SBE: standard base excess.

**Table S2: Comorbidities category codes from ICD-10-CM.**

| Category | ICD-10-codes |
| --- | --- |
| Cardiovascular disease | “I50”,”I20”, ”I21” , ”I22” , ”I23”, ”I24” , ”I25” , ”I15” , ”I70” , ”I97” |
| Hypertension | ”I10” , ”I11” , ”I12” , ”I13”, ”I14” , |
| Liver disease | “K71”, “K72”, “K73”, “K74”, “K75”, “K76”, “K77”, “R93.2” |
| Digestive disease | “K25”,”K26”,“K27”,“K28”,“K29”,”K63”,”K31.6”,”K63.2”,” K51”,”K86.8” |
| Diabetes | “E10”, “E11” |
| Kidney disease | “N18”,”N19” |
| Pulmonary disease | “J44”,”R91” |

**Table S3: Missing rate for demographics and clinical variables extracted from the database**

| Variable | Number of missing | Percent of missing (%) |
| --- | --- | --- |
| Age | 0 | 0 |
| Gender | 0 | 0 |
| Smoke | 0 | 0 |
| Drink | 0 | 0 |
| BMI | 0 | 0 |
| Body temperature | 0 | 0 |
| Heart rate | 0 | 0 |
| Respiratory rate | 0 | 0 |
| SBP | 0 | 0 |
| DBP | 0 | 0 |
| SOFA score | 0 | 0 |
| APACHE-II score | 0 | 0 |
| APS-III score | 0 | 0 |
| Cardiovascular disease | 0 | 0 |
| Hypertension | 0 | 0 |
| Liver disease | 0 | 0 |
| Digestive disease | 0 | 0 |
| Diabetes | 0 | 0 |
| Kidney disease | 0 | 0 |
| Pulmonary disease | 0 | 0 |
| HB | 0 | 0 |
| WBC | 0 | 0 |
| PLT | 203 | 10.4 |
| Albumin | 5 | 0.3 |
| AST | 6 | 0.3 |
| ALT | 9 | 0.5 |
| DBIL | 14 | 0.7 |
| IBIL | 14 | 0.7 |
| Serum creatinine | 0 | 0 |
| eGFR | 0 | 0 |
| APTT | 5 | 0.3 |
| PT | 5 | 0.3 |
| Fib | 5 | 0.3 |
| CRP | 194 | 9.9 |
| PCT | 99 | 5.1 |
| IL-6 | 233 | 11.9 |
| PaO2 | 41 | 2.1 |
| PaCO2 | 24 | 1.2 |
| ABE | 24 | 1.2 |
| SBE | 31 | 1.6 |
| Lactic acid | 53 | 2.7 |

BMI: body mass index; SBP: systolic blood pressure; DBP: diastolic blood pressure; APS-III score: Acute Physiology III score; SOFA score: Sequential Organ Failure Assessment score; APACHE-II score:Acute Physiology and Chronic Health Evaluation II score; ICU: intensive care unit; WBC: white blood cell; HB: hemoglobin; PLT: platelet; AST: aspartate aminotransferase; ALT: alanine aminotransferase; DBIL: direct bilirubin; IBIL: indirect bilirubin; eGFR: estimated glomerular filtration rate; APTT: activated partial thromboplastin time; PT: prothrombin time; Fib: fibrinogen; CRP: C-reactive protein; PCT: procalcitonin; IL-6: interleukin-6; PaO2: partial pressure of oxygen in arterial blood; PaCO2: Partial Pressure of Carbon Dioxide in Arterial Blood; ABE: actual base excess; SBE: standard base excess.

| Number of  groups | LL | BIC | Null Model | 2△BIC | AIC | Average posterior probabilities (%) | Participants per group (%) |
| --- | --- | --- | --- | --- | --- | --- | --- |
| 1 | -25130.2 | -25149.1 | - | - | -25135.2 | 100 | 100 |
| 2 | -24767.6 | -24805.4 | 1 | 687.4 | -24777.6 | 86.1-83.1 | 59.2-40.8 |
| 3 | -24628.4 | -24685.2 | 2 | 240.4 | -24643.4 | 81.2-84.5-80.7 | 14.6-65.1-20.3 |
| 4 | -24495.9 | -24571.6 | 3 | 227.2 | -24515.9 | 76.3-80.3-81.9-81.3 | 10.4-12.0-59.5-18.1 |
| 5 | -24438.9 | -24533.6 | 4 | 76.0 | -24463.9 | 75.9-80.1-81.9-69.4-76.9 | 9.7-11.0-58.0-10.8-10.5 |

**Table S4: Results of group-based trajectory modeling.**

According to the statistical model, the number of trajectories that provided the lowest LL, BIC, and AIC with acceptable average posterior probabilities (> 0.7) was determined to be 4. Therefore, this was deemed as the most suitable number of trajectories.

LL: Log-likelihood; AIC: Akaike information criterion; BIC: Bayesian information criteria; △BIC: The change in Bayesian information criteria

**Table S5: The analysis of optimal trend for trajectories.**

| Trends of each  Trajectories^#^ | Group | Parameter | Estimate | Standard Error | T for H0^*^ | Prob > \|T\| |
| --- | --- | --- | --- | --- | --- | --- |
| 3 3 3 3 | 1 | Intercept | 24.09898 | 1.73244 | 13.91 | 0.0000 |
|  |  | Linear | 0.2876 | 1.669 | 0.172 | 0.8632 |
|  |  | Quadratic | 0.20066 | 0.45554 | 0.44 | 0.6596 |
|  |  | Cubic | -0.02547 | 0.03689 | -0.691 | 0.4899 |
|  | 2 | Intercept | 1.40676 | 1.61136 | 0.873 | 0.3827 |
|  |  | Linear | 20.09637 | 1.56491 | 12.842 | 0.0000 |
|  |  | Quadratic | -4.0407 | 0.43104 | -9.374 | 0.0000 |
|  |  | Cubic | 0.26231 | 0.03527 | 7.437 | 0.0000 |
|  | 3 | Intercept | 28.29236 | 0.60146 | 47.039 | 0.0000 |
|  |  | Linear | 1.54523 | 0.59307 | 2.605 | 0.0092 |
|  |  | Quadratic | -0.16121 | 0.16559 | -0.974 | 0.3303 |
|  |  | Cubic | 0.00399 | 0.01369 | 0.291 | 0.7711 |
|  | 4 | Intercept | 32.00465 | 1.14016 | 28.07 | 0.0000 |
|  |  | Linear | 1.7339 | 1.09378 | 1.585 | 0.1130 |
|  |  | Quadratic | 0.00061 | 0.30572 | 0.002 | 0.9984 |
|  |  | Cubic | -0.01765 | 0.02526 | -0.699 | 0.4848 |
| 1 2 2 2 | 1 | Intercept | 24.73712 | 0.58312 | 42.422 | 0 |
|  |  | Linear | 0.37999 | 0.11866 | 3.202 | 0.0014 |
|  | 2 | Intercept | 10.64016 | 0.9043 | 11.766 | 0.0000 |
|  |  | Linear | 9.40621 | 0.49385 | 19.047 | 0.0000 |
|  |  | Quadratic | -0.90785 | 0.05841 | -15.544 | 0.0000 |
|  | 3 | Intercept | 28.48562 | 0.38126 | 74.714 | 0.0000 |
|  |  | Linear | 1.36773 | 0.20441 | 6.691 | 0.0000 |
|  |  | Quadratic | -0.11329 | 0.02378 | -4.764 | 0.0000 |
|  | 4 | Intercept | 31.13276 | 0.63644 | 48.917 | 0.0000 |
|  |  | Linear | 2.5359 | 0.34422 | 7.367 | 0.0000 |
|  |  | Quadratic | -0.21779 | 0.04102 | -5.309 | 0.0000 |

#: Each number represents the type of trend for a corresponding trajectory. The value "1" indicates a linear trend for the corresponding trajectory, whereas the value "2" and "3" indicates a quadratic trend and a cubic trend, respectively.

*: H0 : parameter = 0

**T****ableS6-1：Multivariable Cox regression analysis for** **different Albumin trajectory groups and 28-day mortality**

| **Alb Group** | **Model 1** | | **Model 2** | |
| --- | --- | --- | --- | --- |
|  | **HR(95%CI)** | **P value** | **HR(95%CI)** | **P value** |
| Group1 | 1 | Reference | 1 | Reference |
| Group2 | 0.52(0.38-0.71) | <0.001 | 0.51(0.37-0.69) | <0.001 |
| Group3 | 0.61(0.49-0.77) | <0.001 | 0.64(0.51-0.81) | <0.001 |
| Group4 | 0.55(0.42-0.72) | <0.001 | 0.62(0.47-0.82) | <0.001 |
|  |  |  |  |  |
| Group2 | 1 | Reference | 1 | Reference |
| Group3 | 1.18(0.94-1.49) | 0.163 | 1.23(0.97-1.57) | 0.086 |
| Group4 | 1.04(0.80-1.38) | 0.738 | 1.20(0.90-1.59) | 0.217 |
|  |  |  |  |  |
| Group3 | 1 | Reference | 1 | Reference |
| Group4 | 0.89(0.74-1.07) | 0.215 | 0.97(0.80-1.17) | 0.732 |

Model 1: Unadjusted rough model. Model 2: Fully adjusted model(adjusted for variables from DAG)

**TableS6-2：Multivariable Cox regression analysis for alb group and 28-day mortality**

| Variables | Model 1 | | Model 2 | | Model 3 | |
| --- | --- | --- | --- | --- | --- | --- |
|  | HR(95%CI) | P value | HR(95%CI) | P value | HR(95%CI) | ***P* value** |
| Alb Group |  |  |  |  |  |  |
| Group1 | 1 | Reference | 1 | Reference | 1 | Reference |
| Group2 | 0.52(0.38-0.71) | <0.001 | 0.51(0.37-0.69) | <0.001 | 0.48(0.35-0.65) | <0.001 |
| Group3 | 0.61(0.49-0.77) | <0.001 | 0.64(0.51-0.81) | <0.001 | 0.69(0.55-0.87) | 0.002 |
| Group4 | 0.55(0.42-0.72) | <0.001 | 0.62(0.47-0.82) | <0.001 | 0.66(0.50-0.88) | 0.005 |
| Covariates |  |  |  |  |  |  |
| Age > 65 (years) |  |  | 1.43(1.25-1.64) | <0.001 | 1.02(0.84-1.23) | <0.001 |
| Gender male |  |  | 1.08(0.93-1.26) | 0.299 | 1.05(0.90-1.22) | 0.002 |
| BMI ≥ 25 (kg/m^2^) |  |  | 1.01(0.88-1.16) | 0.918 | 1.03(0.89-1.20) | 0.005 |
| Renal disease |  |  | 1.22(0.90-1.66) | 0.200 | 1.02(0.74-1.40) | 0.852 |
| Digestive System Disease |  |  | 1.41(1.23-1.62) | <0.001 | 1.31(1.14-1.51) | 0.557 |
| Liver disease |  |  | 1.07(0.87-1.31) | 0.521 | 0.95(0.77-1.19) | 0.650 |
| Alb infusion > 100 (g/first week) |  |  | 1.26(1.10-1.45) | 0.001 | 1.05(0.90-1.22) | 0.908 |
| Cardiovascular disease |  |  |  |  | 1.24(1.01-1.53) | <0.001 |
| Diabetes |  |  |  |  | 1.03(0.88-1.21) | 0.676 |
| Hypertension |  |  |  |  | 1.06(0.90-1.24) | 0.523 |
| Body temperature(T℃) |  |  |  |  | 0.87(0.75-1.00) | 0.044 |
| Heart rate(beats/min) |  |  |  |  | 1.03(0.89-1.19) | 0.719 |
| Respiratory rate (beats/min) |  |  |  |  | 0.94(0.82-1.08) | 0.483 |
| SBP (mmHg) |  |  |  |  | 0.92(0.79-1.07) | 0.057 |
| DBP(mmHg) |  |  |  |  | 1.09(0.93-1.27) | 0.690 |
| SOFA score |  |  |  |  | 1.04(1.01-1.06) | 0.378 |
| APACHE-II score |  |  |  |  | 1.07(1.04-1.10) | 0.259 |
| APS-III score |  |  |  |  | 0.95(0.92-0.98) | 0.277 |
| WBC (*10^9^/L) |  |  |  |  | 1.03(0.89-1.18) | 0.010 |
| PLT (*10^9^/L) |  |  |  |  | 1.06(0.90-1.24) | <0.001 |
| AST (U/L) |  |  |  |  | 1.13(0.98-1.31) | 0.002 |
| DBIL (μmol/L) |  |  |  |  | 0.97(0.82-1.15) | 0.704 |
| IBIL (μmol/L) |  |  |  |  | 0.91(0.78-1.05) | 0.497 |
| CRP (mg/L) |  |  |  |  | 0.87(0.73-1.03) | 0.091 |
| PCT (ng/ml) |  |  |  |  | 0.96(0.81-1.14) | 0.729 |
| IL-6 (pg/ml) |  |  |  |  | 1.22(1.04-1.42) | 0.196 |
| Lactic acid (mmol/L) |  |  |  |  | 1.15(0.99-1.33) | 0.111 |
| HB (g/L) |  |  |  |  | 0.99(0.86-1.14) | 0.674 |
| eGFR (ml/min/1.73 m^2^) |  |  |  |  | 0.82(0.70-0.97) | 0.015 |
| APTT(s) |  |  |  |  | 1.13(0.96-1.34) | 0.064 |
| PT(s) |  |  |  |  | 1.14(0.97-1.35) | 0.877 |
| Fib(g/L) |  |  |  |  | 0.99(0.84-1.16) | 0.024 |
| PaCO2 (mmHg) |  |  |  |  | 1.04(0.90-1.21) | 0.146 |
| ABE (mmol/L) |  |  |  |  | 1.51(0.97-2.34) | 0.111 |
| SBE (mmol/L) |  |  |  |  | 0.76(0.49-1.19) | 0.892 |

Model 1: Unadjusted rough model.

Model 2: Adjusted for Age, Gender, BMI, Comorbidities: Cardiovascular Disease, Renal disease, Digestive System Disease, Liver diseases, Alb infusion in the first week based on the result of Directed Acyclic Graph.

Model 3: Fully adjusted Cox regression model; adjusted for (1) baseline characteristics at admission: Age, Gender, BMI, Body temperature, Heart rate, Respiratory rate, SBP, DBP, SOFA score, APACHE-II score, APS-III score, (2) Comorbidities: Cardiovascular disease, Hypertension, Liver disease, Digestive disease, Diabetes, Renel disease, (3) laboratory test results: HB, WBC, PLT, Alb, AST, DBIL, IBIL, eGFR, APTT, PT, Fib, CRP, PCT,IL-6, PaCO3, ABE, SBE, Lactic acid.

BMI: body mass index; SBP: systolic blood pressure; DBP: diastolic blood pressure; APS-III score: Acute Physiology III score; SOFA score: Sequential Organ Failure Assessment score; APACHE-II score:Acute Physiology and Chronic Health Evaluation II score; ICU: intensive care unit; WBC: white blood cell; HB: hemoglobin; PLT: platelet; AST: aspartate aminotransferase; ALT: alanine aminotransferase; DBIL: direct bilirubin; IBIL: indirect bilirubin; eGFR: estimated glomerular filtration rate; APTT: activated partial thromboplastin time; PT: prothrombin time; Fib: fibrinogen; CRP: C-reactive protein; PCT: procalcitonin; IL-6: interleukin-6; PaO2: partial pressure of oxygen in arterial blood; PaCO2: Partial Pressure of Carbon Dioxide in Arterial Blood; ABE: actual base excess; SBE: standard base excess.

**TableS6-3：Multivariable Cox regression analysis for alb group and 28-day mortality**

| Variables | Model 1 | | Model 2 | |
| --- | --- | --- | --- | --- |
|  | HR(95%CI) | P value | HR(95%CI) | P value |
| Alb Group |  |  |  |  |
| Group2 | 1 | Reference | 1 | Reference |
| Group3 | 1.18(0.94-1.49) | 0.163 | 1.23(0.97-1.57) | 0.086 |
| Group4 | 1.04(0.80-1.38) | 0.738 | 1.20(0.90-1.59) | 0.217 |
| Covariates |  |  |  |  |
| Age > 65 (years) |  |  | 1.58(1.36-1.83) | <0.001 |
| Gender male |  |  | 1.02(0.87-1.18) | 0.839 |
| BMI ≥ 25 (kg/m^2^) |  |  | 1.05(0.82-1.33) | 0.707 |
| Renal disease |  |  | 1.18(0.87-1.62) | 0.291 |
| Digestive System Disease |  |  | 1.44(1.24-1.66) | <0.001 |
| Liver disease |  |  | 1.20(0.97-1.48) | 0.090 |
| Alb infusion > 100 (g/first week) |  |  | 1.30(1.12-1.50) | <0.001 |

Model 1: Unadjusted rough model. Model 2: Fully adjusted model(adjusted for variables from DAG)

**TableS6-4：Multivariable Cox regression analysis for alb group and 28-day mortality**

| Variables | Model 1 | | Model 2 | |
| --- | --- | --- | --- | --- |
|  | HR(95%CI) | P value | HR(95%CI) | P value |
| Alb Group |  |  |  |  |
| Group3 | 1 | Reference | 1 | Reference |
| Group4 | 0.89(0.74-1.07) | 0.215 | 0.97(0.80-1.17) | 0.732 |
| Covariates |  |  |  |  |
| Age > 65 (years) |  |  | 1.54(1.32-1.80) | <0.001 |
| Gender male |  |  | 1.05(0.90-1.24) | 0.514 |
| BMI ≥ 25 (kg/m^2^) |  |  | 0.99(0.77-1.28) | 0.947 |
| Renal disease |  |  | 1.20(0.87-1.65) | 0.267 |
| Digestive System Disease |  |  | 1.41(1.21-1.64) | <0.001 |
| Liver disease |  |  | 1.18(0.95-1.48) | 0.137 |
| Alb infusion > 100 (g/first week) |  |  | 1.33(1.14-1.55) | <0.001 |

Model 1: Unadjusted rough model. Model 2: Fully adjusted model(adjusted for variables from DAG)

**Figure S1: Directed acyclic graph (DAG) illustrating the potential actions of confounding covariates on the relation between different Albumin trajectory patterns and clinical outcomes.**


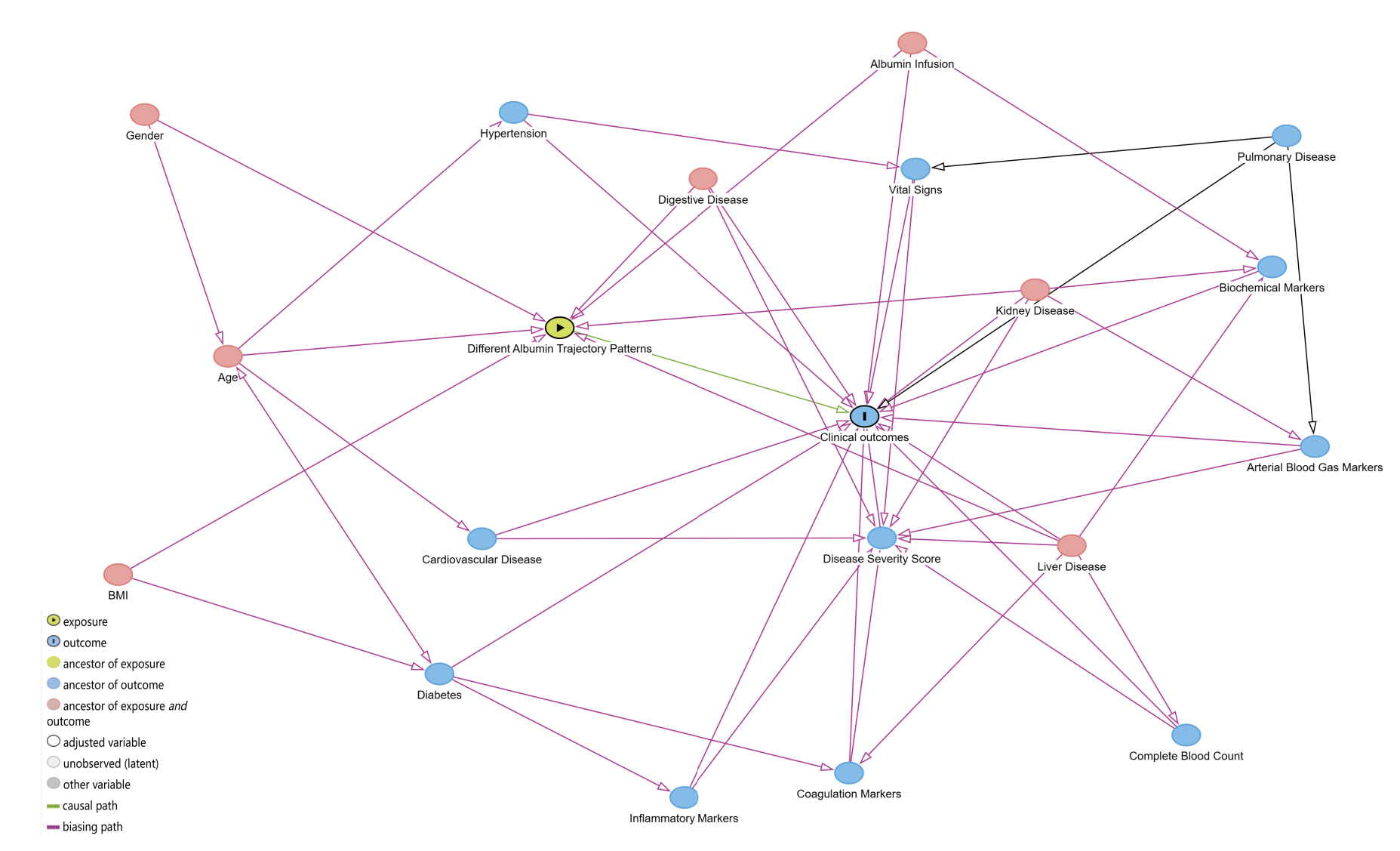


BMI: body mass index

**Figure S2: The incidence of adverse events in each Alb trajectory group.**


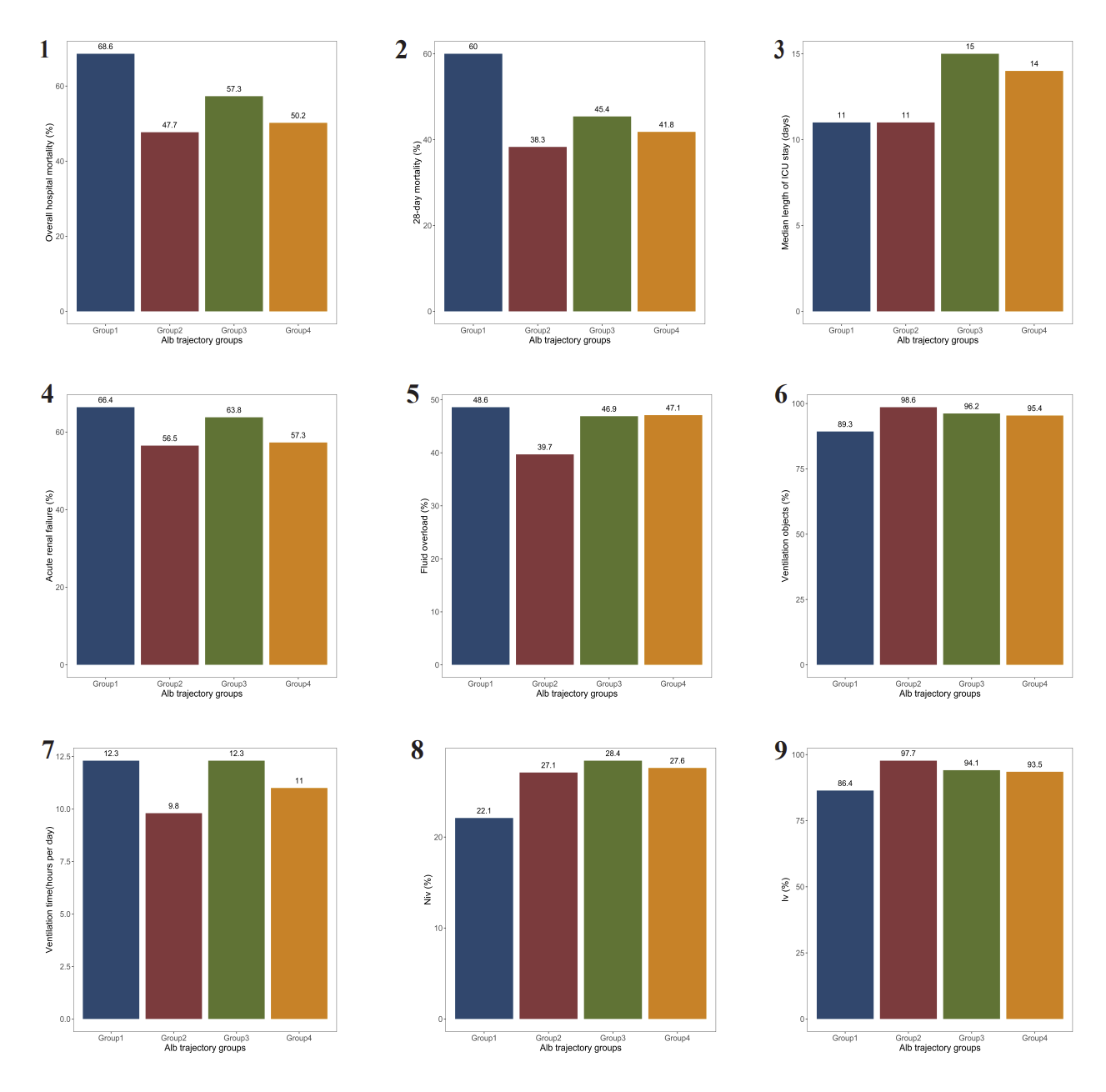

Supplement: Supplementary file 1 [file Data_Sheet_1.docx]
